# Supplementary material for: An ALS assembly modulator signature in peripheral blood mononuclear cells: implications for ALS pathophysiology, therapeutics, and diagnostics
Source: Clin Proteomics. 2025 Apr 28;22:16. doi: 10.1186/s12014-025-09538-4 (PMC12036218; doi:10.1186/s12014-025-09538-4)
Supplement: Supplementary file 1 — Supplementary material 1. [file 12014_2025_9538_MOESM1_ESM.docx]

**Supplemental Figure 2.**

Legend to Supplemental Figure 2. Spectral counts for entire dataset of MoBr and PBMC THIQ resin eluates. Colors represent the number of spectral counts for a given protein in each sample, ranging from light green (low counts) to dark green (high counts). The right panel indicates whether a given protein was in one of both of the datasets, according to threshold values of >4 spectral counts in the drug eluate and <4 spectral counts in the control column eluate.

**Supplemental Figure 3.**

Legend to Supplemental Figure 3. Confirmation that only a small subset of PDI is present in the DRAC-identified target and that very little additional pre-existing PDI is competent for formation of new target without *de novo* synthesis. PBMC extracts were prepared as previously, applied to drug resins and bound proteins analyzed by WB for PDI. The band corresponding to the correct molecular weight was quantified as shown. Samples from three ALS patients and one healthy control are shown. Column 459 represents the THIQ drug resin and column 134 the control drug resin. As can be seen, approximately 10% of PDI is in the complex bound to the drug resin in all cases, with no significant binding to the control resin in any case. The flowthrough from the first column (drug or control) was applied to a second copy of the drug resin, demonstrating substantial depletion from the first column and that, despite losses, a substantial fraction of the flow through from the control column remains competent to bind to the drug resin column.
